# Supplementary material for: DNA Methylation and Hydroxymethylation Quantification Using Vibrational Spectroscopy
Source: ACS Meas Sci Au. 2026 Jul 24;6(5):1724–38. doi: 10.1021/acsmeasuresciau.6c00167 (PMC13693256; doi:10.1021/acsmeasuresciau.6c00167)
Supplement: Supplementary file 1 [file tg6c00167_si_001.pdf]

Supporting Information

DNA Methylation and Hydroxymethylation Quantification using Vibrational Spectroscopy

Rashad Fatayer[1], Stephen-John Sammut[2,3*], Ganapathy Senthil Murugan[1*]

[1]Optoelectronics Research Centre, Building 53, University of Southampton, University Road, Southampton, Hampshire, SO17 1BJ, United Kingdom

[2] The Breast Cancer Now Toby Robins Research Centre, The Institute of Cancer Research, Mary-Jean Mitchell Green Building, Chester Beatty Laboratories, 237 Fulham Road, London, SW3 6JB, United Kingdom

[3] The Royal Marsden NHS Foundation Trust, Fulham Road, London, SW3 6JJ, United Kingdom

** To whom correspondence can be addressed to: smg@orc.soton.ac.uk, stephen-john.sammut@icr.ac.uk*

Supporting Table Legends:

Supporting Table 1: The 338 base-pair sequence of the *APC* gene used to generate the double stranded DNA standards used in the study.

Supporting Table 2: Mixture compositions for the independent 5-mC and 5-hmC *APC* DNA percentage series (0-100% in 20% increments), prepared by mixing unmethylated DNA with fully methylated or fully hydroxymethylated standards at constant total DNA concentration and volume.

Supporting Table 3: Composition of simplex-based global cytosine modification mixtures, including binary 5-mC/5-hmC standards (no unmethylated DNA) and ternary mixtures formed by adding unmethylated *APC* DNA; all mixtures were prepared at constant total DNA concentration and final volume.

Supporting Table 4: Composition of the ctDNA methylation mixtures prepared by proportionally mixing methylated and unmethylated reference materials (*CCND2, EGFR, ETV6, FANCA, MYB, RET, TFRC*) to generate target global methylation levels (0-100, in 20% increments) at constant DNA concentration (10 ng/µL) and final volume, using manufacturer-specified regional methylation values averaged across loci to define global reference levels.

Supporting Figures

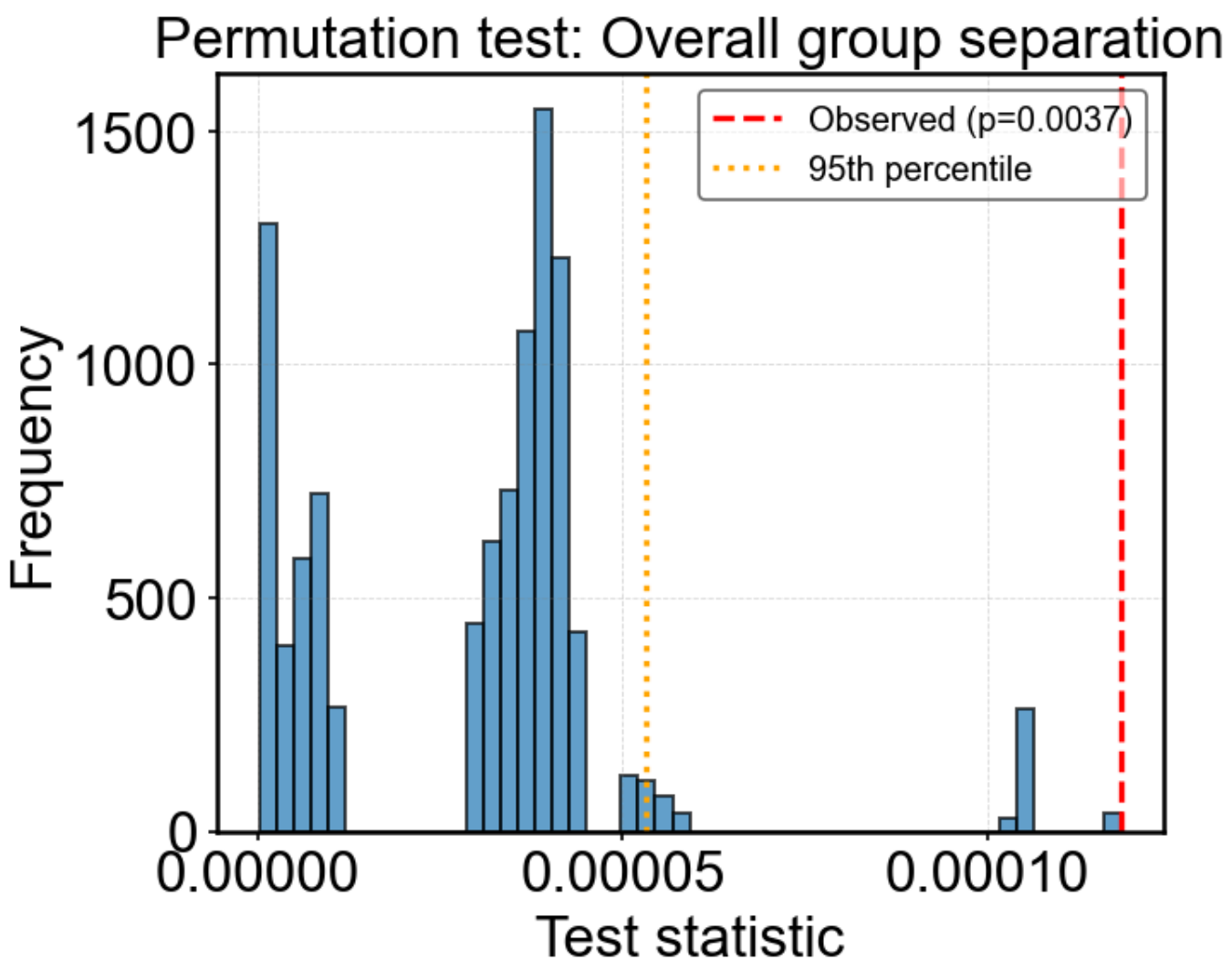

Supporting Figure 1: Permutation test for overall PCA group separation of unmethylated, 100% 5-mC, and 100% 5-hmC DNA. The histogram shows the empirical null distribution of the between-group variance statistic T obtained from 10,000 random permutations of group labels across all sample replicates in PCA latent space. The red dashed line indicates the observed statistic T_{obs} computed on the true group assignments. The orange dotted line marks the 95th percentile of the null distribution. The observed statistic falls far beyond the null distribution, yielding $p = 0.0037$, indicating that the separation of the three groups in PCA space is highly unlikely to have arisen by chance.

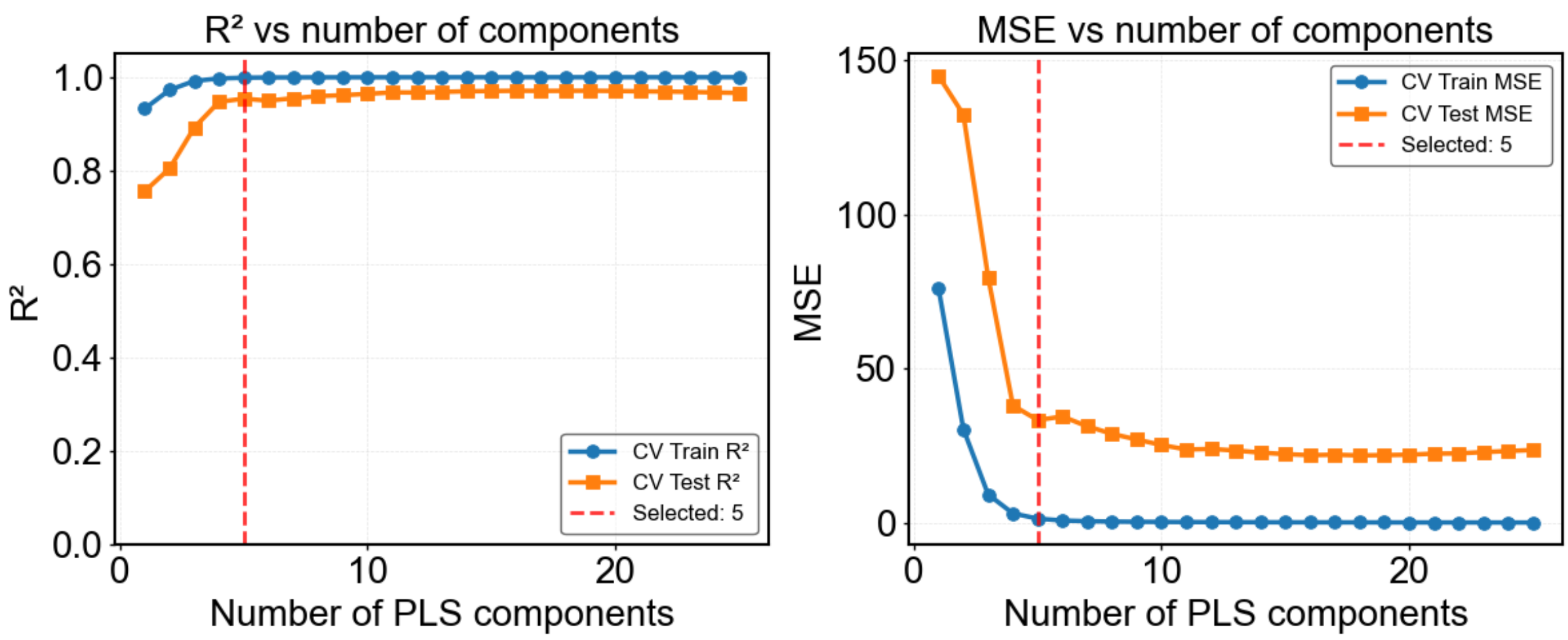

Supporting Figure 2: Optimisation of the number of latent variables (LVs) for PLSR models of individually changing methylation percentage DNA samples, evaluated using the coefficient of determination (R^2) and mean squared error (MSE). The optimal LV number was determined by cross-validation on the CV train/test split, and model significance was assessed using Van der Voet's F-test.

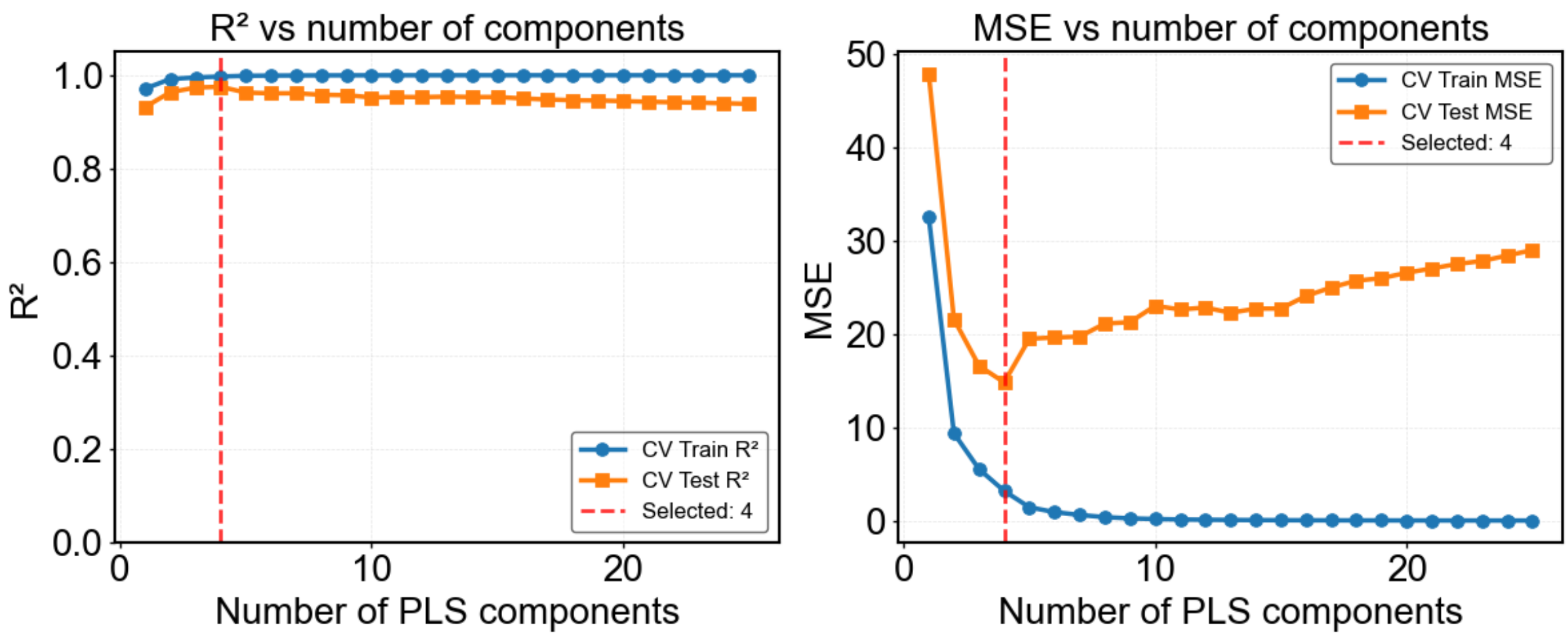

Supporting Figure 3: Optimisation of the number of LVs for PLSR models of individually changing hydroxymethylation percentage DNA samples, evaluated using R^2 and MSE.

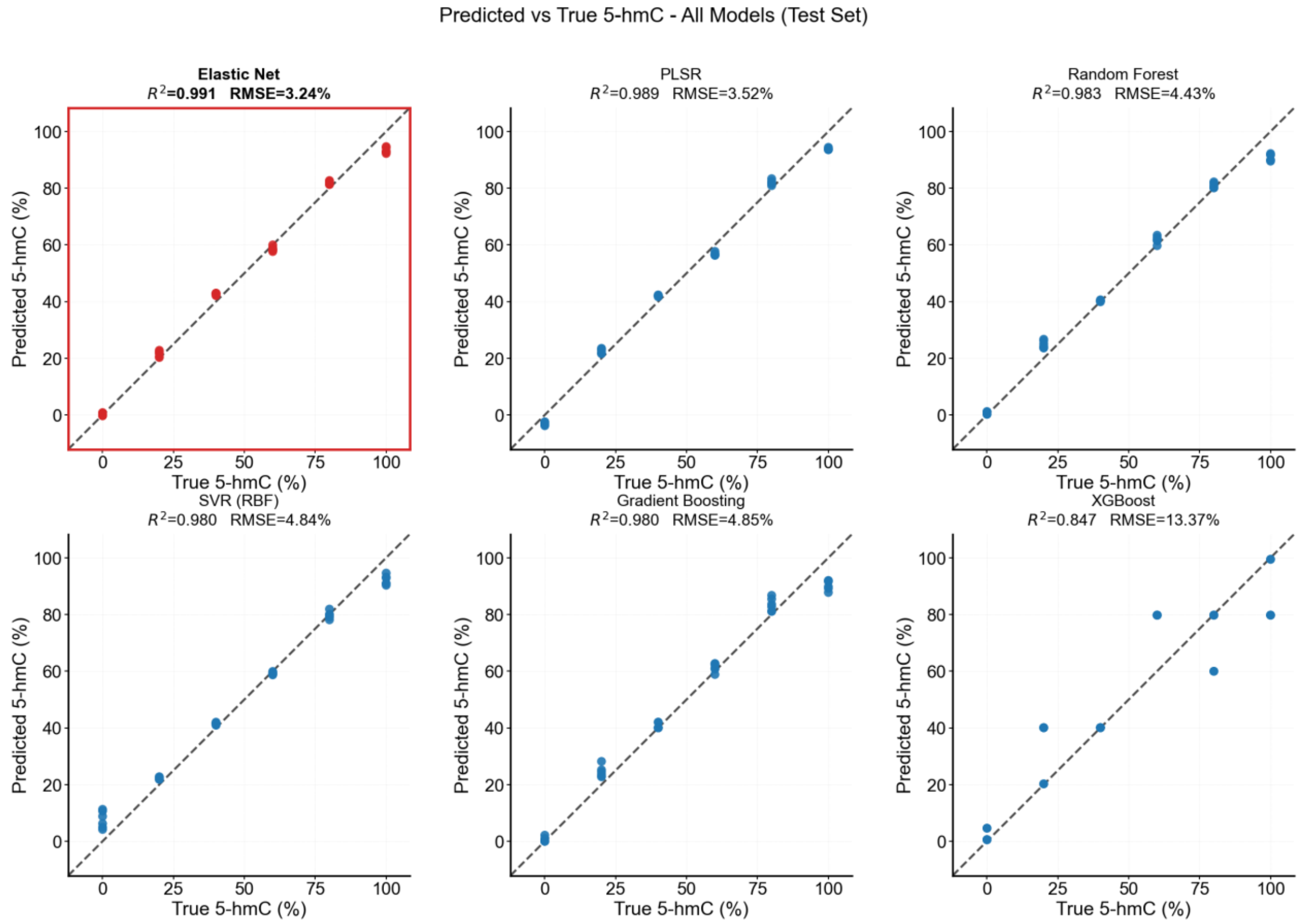

Supporting Figure 4: Predicted versus true 5-hmC (%) for all evaluated regression models on the held-out test set, ranked by test R^2. The best-performing model (Elastic Net, R^2=0.991, RMSE=3.24%) is highlighted with a red border. All models were tuned via GridSearchCV with group-wise cross-validation (GroupKFold, k=5), ensuring replicate spectra were excluded from validation folds during hyperparameter selection. PLSR achieved comparable accuracy (R^2=0.988, RMSE=3.52%).

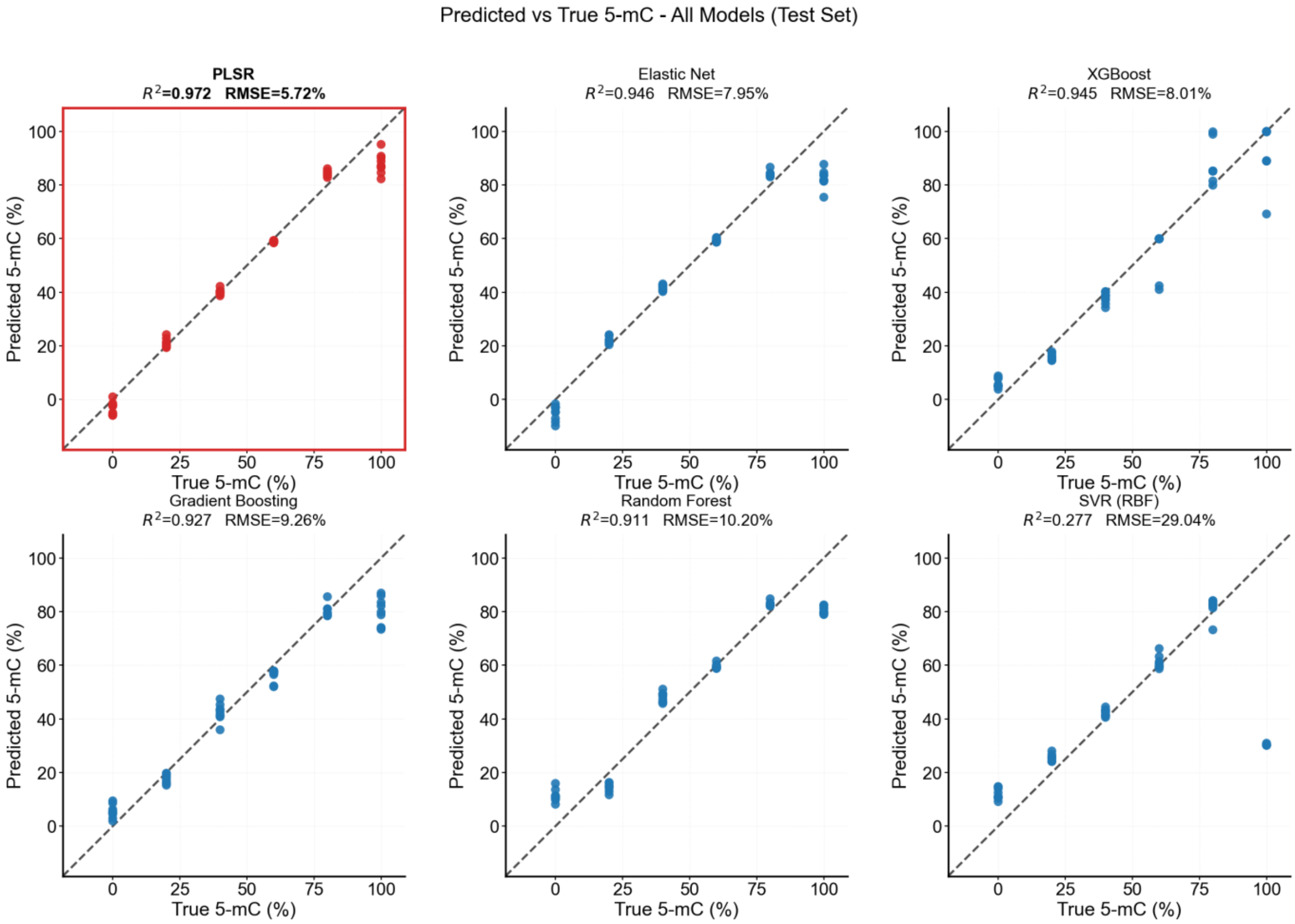

Supporting Figure 5: Predicted versus true 5-mC (%) for all evaluated regression models on the held-out test set, ranked by test R^2. PLSR was the best-performing model (R^2=0.972, RMSE=5.72%, highlighted with a red border), outperforming Elastic Net (R^2=0.946, RMSE=7.95%), XGBoost (R^2=0.945, RMSE=8.01%), Gradient Boosting (R^2=0.927, RMSE=9.26%), Random Forest (R^2=0.911, RMSE=10.20%), and SVR (RBF) (R^2=0.277, RMSE=29.04%). All models were tuned via GridSearchCV with group-wise cross-validation (GroupKFold, k=5), ensuring replicate spectra were excluded from validation folds during hyperparameter selection.

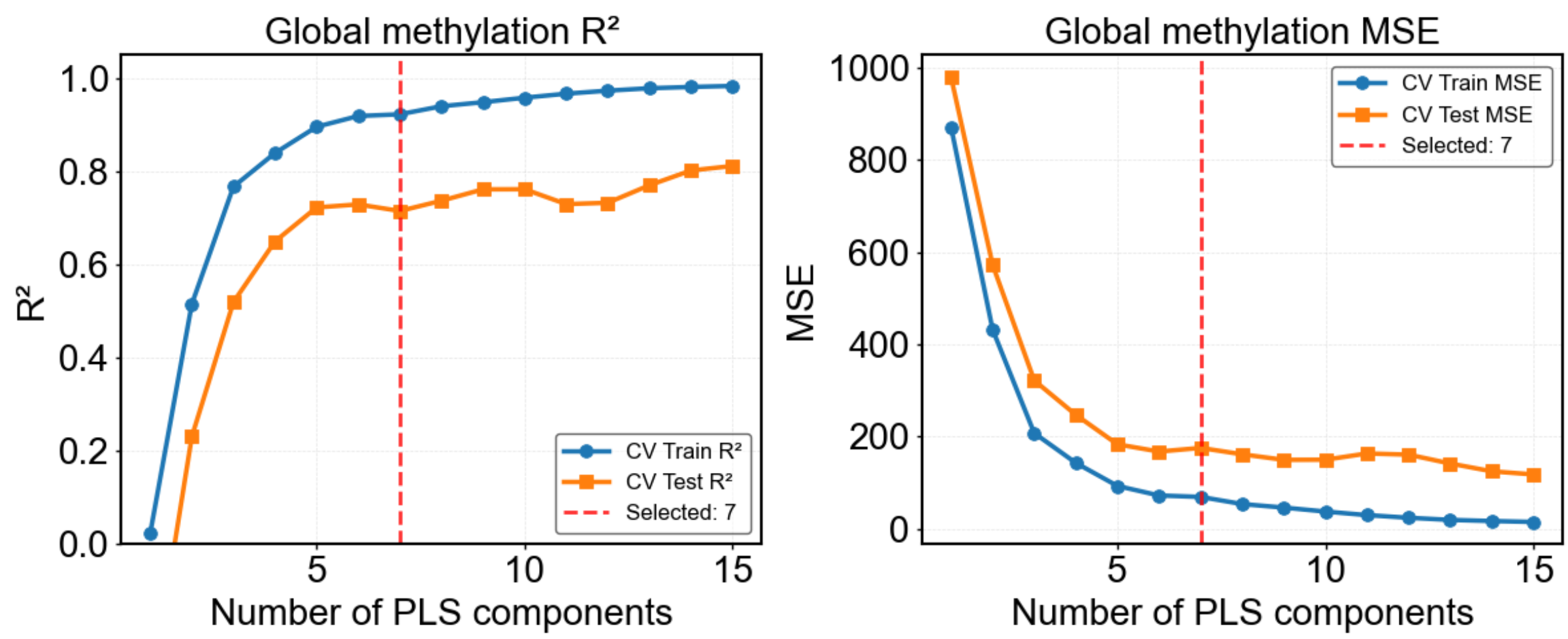

Supporting Figure 6: Optimisation of the number of LVs for PLSR models of globally changing methylation percentage DNA samples, evaluated using R^2 and MSE.

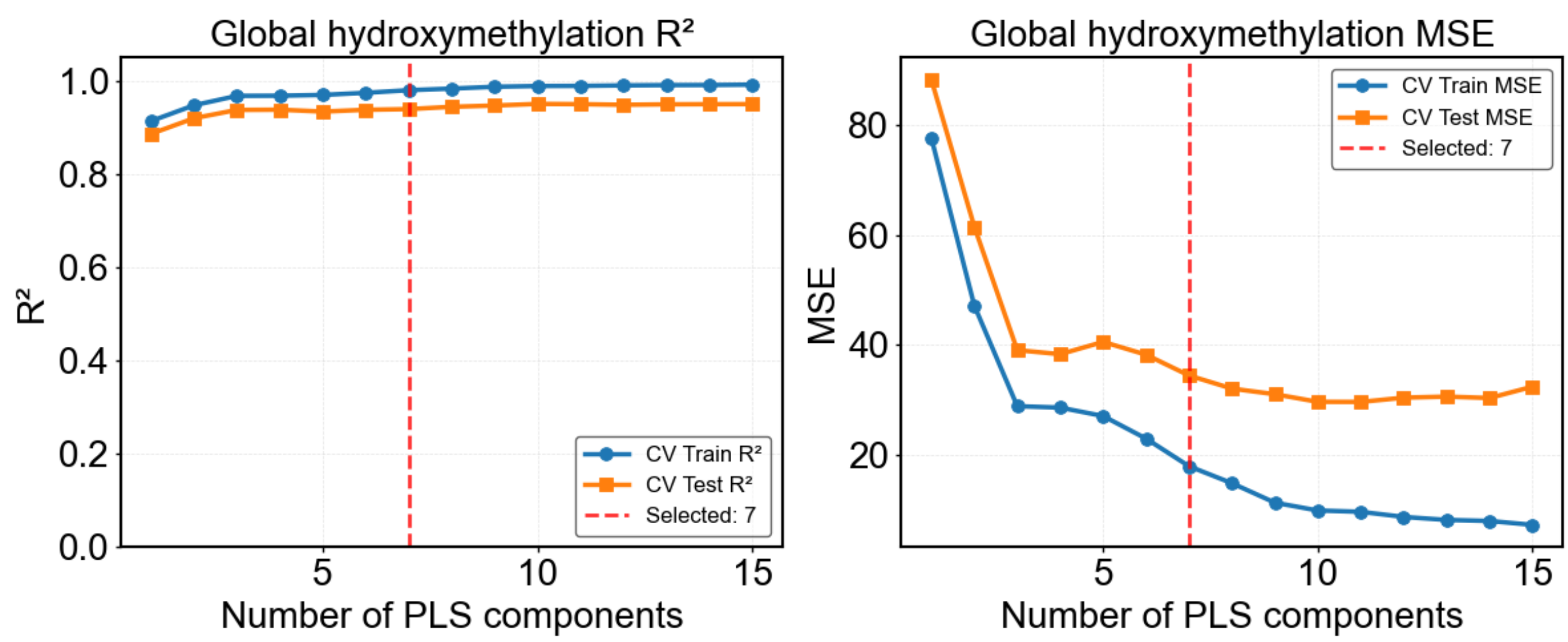

Supporting Figure 7: Optimisation of the number of LVs for PLSR models of globally changing hydroxymethylation percentage DNA samples, evaluated using R^2 and MSE.

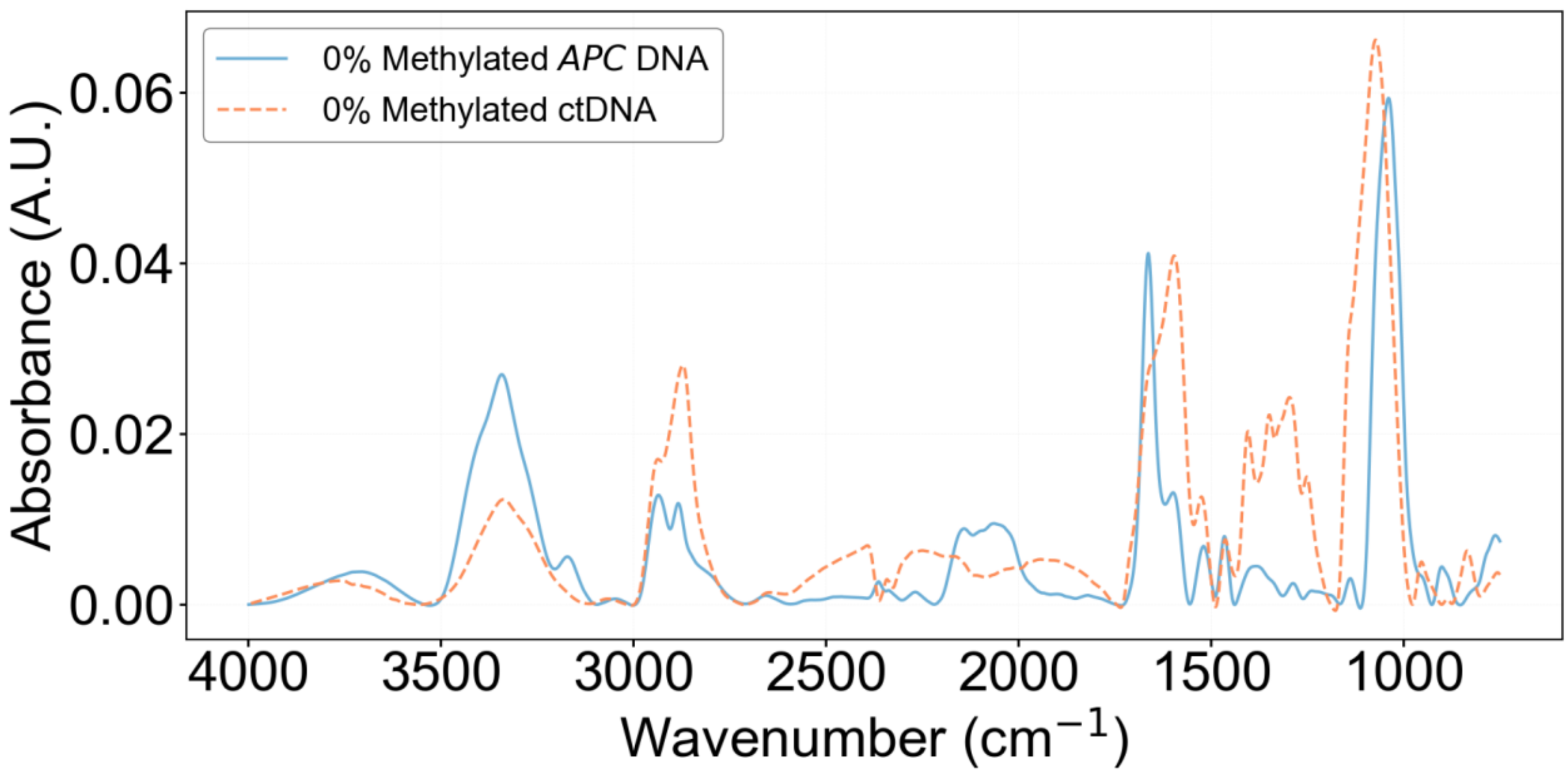

Supporting Figure 8: Comparison of ATR-FTIR spectra of unmethylated *APC* DNA and the unmethylated ctDNA reference standard.
